# The International Medical Congress, Held in London, August 3–9, 1881

**Published:** 1881-10

**Authors:** 


### Societies.

---

### THE INTERNATIONAL MEDICAL CONGRESS.

Held in London, England, August 3–9, 1881.

The seventh meeting of the International Medical Congress was formerly opened in St. James's Hall, by his Royal Highness the Prince of Wales. The members of the Congress began to assemble soon after 10 A.M., and by 11 o'clock every part of the great hall appeared to be filled, nearly 3,000 members of the Congress being present. No ladies were allowed to attend in a professional capacity. This determination of the general committee to exclude ladies who are members of the profession was protested against, and a request that the committee would reconsider their decision, signed by forty-three duly qualified medical women. The protest had no effect.

The Prince of Wales on his arrival was received by Sir William Jenner, Sir William Gull, Sir James Paget, Mr. MacCormac, and other members of the committee, and conducted to the platform. A little later the Crown Prince of Prussia entered and took a seat on the left of the chairman.

Sir William Jenner, K. C. B., President of the Royal College of Physicians, as chairman *ex-officio* of the general committee, took the chair until the President-elect of the Congress had been formerly installed. In opening the proceedings, Sir William Jenner offered the acknowledgments of the medical profession to the Prince of Wales for the support which his Royal Highness had accorded to the Congress, and then mentioned some of the objects which i was sought to promote by that international gathering.

Mr. MacCormac, the honorary secretary-general, next read the report of the executive committee, in which he stated, that altogether more than 120,000 circular notices of the meeting had been sent out.

Surgeon J. S. Billings, for the War Department, and Surgeon Brown, of the Navy Department, were the duly commissioned representatives of the United States.

Among the foreign Vice-Presidents (American) were: Dr. Fordyce Barker, Dr. J. S. Billings, Dr. Bigelow and Dr. Austin Flint.

In addition to those legally qualified to practice medicine in their respective countries, a certain number of home and foreign pharmacists of distinction had been invited as extraordinary members, and also some eminent dentists and physiologists who were not medical men.

After the reading of the report, Sir James Paget, amid loud cheering, took his seat as president of the congress.

The Prince of Wales, who was greeted with loud cheers on rising to speak, said: "Your Imperial Highness and gentlemen—I gladly complied with the request that I should be patron of the International Medical Congress of 1881, and among many reasons for so doing was my conviction that few things can tend more to the welfare of mankind than that educated men of all nations should from time to time meet together for the promotion of the branches of knowledge to which they devote themselves. The intercourse and the mutual esteem of nations have often been advanced by great international exhibitions, and I look back with pleasure to those with which I have been connected; but when conferences are held among those who in all parts of the world apply themselves to the study of science, even greater international benefits may, I think, be confidently anticipated, more especially in the study of medicine and surgery, for in these the effects of climate and of national habits must give to the practitioners of each nation opportunities, not only of acquiring knowledge, but of imparting knowledge to those of their *confreres* whom they meet in Congress. I venture to think, gentlemen, that the Executive Committee have acted wisely in instituting sections for the discussion of a very wide range of subjects, including not only the sciences on which medical knowledge is founded, but many of its most practical applications, and I am very happy to see that so great scope will be granted for the discussion of important questions relating to the public health, to the cure of the sick in hospitals and in the houses of the poor, and to the welfare of the army and navy. The devotion with which many members of the medical profession readily share the dangers of climate and the fatigues and dangers of war, and the many risks which must be encountered in the study of means, not only for the remedy, but for the prevention of disease, deserves the warmest acknowledgment from the public.

The list of officers of the Congress, including as it does the names of those distinguished in every branch of medical science, shows how heartily the proposal to hold the meeting in London has been received. I think it speaks well for the good feeling of the profession that there should have been so warm a response to the invitations. How cordially the proposal has been received may be seen not only in the large number of visitors but in the fact that they include a large proportion of those who enjoy a high reputation not only in their own countries, but throughout the world. I sincerely congratulate the reception committee on this good promise of complete success, and I trust that at the close of the Congress they will feel rewarded for the labor they have bestowed upon it. The report which the Secretary-General, Mr. MacCormac, has read will have explained how great have been his labors. He will hereafter be well repaid, and I am sure Mr. MacCormac is sensible that he will be recompensed even for his great exertions by the assurance that the progress of the important science of medicine has been materially promoted, for any addition to the knowledge of medicine must always be followed by an increase in the happiness of mankind. (Loud cheers.)

Sir James Paget then proceeded to deliver his inaugural address. In the course of which he says: "Although the programme tells of selected subjects for discussion, and defines the order of our work, yet knowledge will be promoted in a much wider range in the meetings without order, which will be held every day and everywhere—meetings of men with all kinds of mental power and all forms of knowledge and of skill; everyone ready alike to impart and acquire knowledge. It is safe to say that, in the casual conversations of the coming week, there will be a larger interchange and diffusion of information than in any equal time and space in the whole past history of medicine. And with this interchange will be a larger increase, for, in the mart of knowledge he that receives gains, and he that gives retains, and none suffer loss. The increase will be the greater because of the great variety of minds which will meet. As I look round this hall, my admiration is moved, not only by the number and total power of the minds which are here, but by their diversity—a diversity in which, I believe, they fairly represent the whole of those who are engaged in the cultivation of our science. For here are minds representing the distinctive characters of all the most gifted and most educated nations—characters still distinctly national in spite of the constantly increasing intercourse of the nations. And from many of these nations we have both elder and younger men; thoughtful men and practical; men of fact and men of imagination; some confident, some sceptic; various, also, in education, in purpose and mode of study, in disposition and in power.

"It has been said, indeed, that truth is more likely to emerge from error than from confusion, and, in some instances, this is true; but much of what we call confusion is only the order of nature not yet discerned; and so it may be here.

"In that which each man believes that he observes there is something of himself; and for certainty, even on matters of fact, we often need the agreement of many minds, that the personal element of each may be counteracted. And much more is this necessary in the consideration of the many questions which are to be decided by discussing the several values of admitted facts and of probabilities, and of the conclusions drawn from them. For, on questions such as these, minds of all kinds may be well employed. Here there will be occasion even for those which are not unconditionally praiseworthy, such as those that habitually doubt, and those to whom the invention of argument is more pleasing than the mere search for truth. Nay, we may be able to observe the utility even of error. We may not, indeed, wish for a prevalence of errors; they are not more desirable than the crime and misery which evoke charity. And yet, in a congress we may palliate them, for we may see how, as we often read in history, errors, like doubts and contrary pleadings, serve to bring out the truth, to make it express itself in clearest terms and show its whole strength and value. But that which I would chiefly note, in relation to the great variety of minds which are here, is that it is characteristic of that mental pliancy and readiness for variation which is essential to all scientific progress, and which a great International Congress may illustrate and promote.

"Let me speak of the sections to defend them; for some maintain that, even in such a division of studies as these may encourage, there is a mischievous dispersion of forces. The science of medicine, which used to be praised as one and indivisible, is broken up, they say, among specialists, who work in conflict rather than in concert, and with mutual distrust more than mutual help. But let it be observed that the sections which we have instituted are only some of those which are already recognized in many countries in separate societies, each of which has its own place and rules of self-government, and its own literature. And the division has taken place naturally in the course of events which could not be hindered. For the partial separation of medicine, first from the other natural sciences, and now into sections of its own, has been due to the increase of knowledge being far greater than the increase of individual mental power. I do not doubt that the average mental power constantly increases in the successive generations of all well-trained people, but it does not increase so fast as knowledge does, and thus in every science, as well as in our own, a small portion of the whole sum of knowledge has become as much as even large minds can hold and duly cultivate. Many of us must, for practical life, have a fair acquaintance with many parts of our science, but none can hold it all; and for complete knowledge or for research, or for safely thinking out beyond what is known, no one can hope for success unless by limiting himself within the few divisions of the science for which, by nature, or by education, he is best fitted. Thus, our division into sections is only an instance of that division of labor which in every prosperous nation we see in every field of active life and which is always justified by more work better done. Moreover, it cannot be said that in any of our sections there is not enough for a full strong mind to do. If any one will doubt this, let him try his own stremgth in the discussions of several of them. In truth, the fault of specialism is not in narrowness, but in the shallowness and the belief in self-sufficiency with which it is apt to be associated. If the field of any specialty in science be narrow, it can be dug deeply. In science, as in mining, a very narrow shaft, if only it be carried deep enough, may reach the richest stores of weath and find use for all the appliances of scientific art. Not in medicine alone, but in every department of knowledge, some of the grandest results of research and learning, broad and deep, are to be found in monographs on subjects that to the common mind seemed small and trival.

"The dominance of doctrine has promoted the habit of inference, and repressed that of careful observation and induction. It has encouraged that fallacy to which we are all too prone, that we have at length reached an elevated, sure position on which we may rest, and only think and guide. In this way specialism in doctrine or in method of study has hindered the progress of science more than the specialism which has attached itself to the study of one organ or of one method of practice. This kind of specialism may enslave inferior minds; the specialism of doctrine can enchant into mere dreaming those that should be strong and alert in the work of free research.

"The advance of medical knowledge within one's memory is amazing, whether reckoned in the wonders of the science not yet applied, or in practical results in the general lengthening of life, or, which is still better, in the prevention and decrease of pain and misery, and in the increase of working power.

"In the number and intensity of the questions brought before us, we may see something of our responsibility. If we could gather into thought the amounts of misery or happiness, of helplessness or of power for work, which may depend on the answers to all the questions that will come before us, this might be a measure of our responsibility. But we cannot count it; let us imagine it; we cannot even in imagination exaggerate it. Let us bear it always in our mind, and remind ourselves that our responsibility will constantly increase. For, as men become in the best sense better educated, and the influence of scientific knowledge on their moral and social state increases, so, among all sciences there is none of which the influence and, therefore the responsibility will increase more than ours; because none more intimately concerns man's happiness and working power. But, more clearly in the recollections of the Congress, we may be reminded that in our science there may be, or, rather, there really is, a complete community of interest among men of all nations. On all the questions before us we can differ, discuss, dispute, and stand in earnest rivalry; but all consistently with friendship, all with readiness to wait patiently till more knowledge shall decide which is in the right. Let us resolutely hold to this when we are apart; let our internationality be a clear abiding sentiment, to be, as now, declared and celebrated at appointed times, but never to be forgotten; we may, perhaps, help to gain a new honor for science, if we thus suggest that in many more things, if they were as deeply and dispassionately studied, there might be found the same complete identity of international interest as in ours. And then, let us always remind ourselves of the nobility of our calling. I dare to claim for it, that among all the sciences, ours, in the pursuit and use of truth, offers the most complete and consant union of those three qualities which have the greatest charm for pure and active minds—novelty, utility, and charity.

"In every truth attained there is utility either at hand or among the certainties of the future. And this utility is not selfish; it is not in any degree correlative with money-making; it may generally be estimated in the welfare of others better than in our own. Some of us may, indeed, make money and grow rich; but many of those that minister even to the follies and vices of mankind can make much more money than we. In all things costly and vainglorious they would far surpass us if we would compete with them. We had better not compete where wealth is the highest evidence of success; we can compete with the world in the nobler ambition of being counted among the learned and the good who strive to make the future better and happier than the past. And to this we shall attain if, we will remind ourselves that, as in every pursuit of knowledge there is the charm of novelty, and in every attainment of truth utility, so in every use of it there may be charity. I do not mean only the charity which is in hospitals or in the service of the poor, great as is the privilege of our calling in that we may be its chief ministers, but that wider charity which is practiced in a constant sympathy and gentleness, in patience and self-devotion. And it is surely fair to hold that, as in every search for knowledge we may strengthen our intellectual power, so in every practical employment of it we may, if we will, improve our moral nature; we may obey the whole law of Christian love, we may illustrate the highest indunction of scientific philanthrophy. Let us, then, resolve to devote ourselves to the promotion of the whole science, art, and charity, of medicine. Let this resolve be to us as a vow of brotherhood; and may God help us in our work." (Loud cheers.)

The Congress then resolved itself into its various sections.

At a general meeting held in St. James' Hall in the afternoon, Sir James Paget in the chair, Professor Virchow, of Berlin, delivered, in German, an address on the "Value of Pathological Experiment."

This address was an eloquent plea for vivisection. The learned Professor pleaded that vivisectionists should not be regarded as heartless barbarians, but as men who were working to promote the welfare of humanity. The address lasted over an hour and evoked much applause.

The several sections met in the afternoon. Anatomy was presided over by Professor W. H. Flower, physiology by Dr. Michael Foster, pathology and morbid anatomy by Dr. S. Wilks, medicine by Sir W. Gull, diseases of the throat by Dr. G. Johnson, surgery by Mr. J. Eric Erichsen, obstetric medicine and surgery by Dr. A. M'Clintock, diseases of children by Dr. West, mental diseases by Dr. Lockhart Roberston, diseases of the ear by Mr. W. B. Dalby, diseases of the skin by Mr. Erasmus Wilson, diseases of the teeth by Mr. Edwin Saunders, state medicine by Mr. J. Simon, military surgery and medicine by Professor T. Longmore, materia medica and pharmacology by Professor T. R. Fraser, and ophthalmology by Mr. Bowman.

Mr. Eric Erichsen, in his address before the section on surgery, said: "In no department of surgery has a more marked or a more brilliant advance been made of late years than in that which concerns the operative treatment of intra-peritoneal tumors. The establishment of ovariotomy as a recognized surgical operation has now long been a matter of history; but the perfection of safety to which it has of late years been carried by the improvement of its details, has led the way to a vast and rapid extension of operative surgery for the cure or relief of various diseased abdominal organs. In lithotrity it is probable that a great and real advance has been made. Certainly it is undoubted that a complete revolution has been effected by the enterprise and skill of one of our American brethren, for it cannot be questioned that "Bigelow's operation" has completely changed the aspect of lithotrity, and there is every reason to believe that it constitutes one of those real advances in a method which marks an epoch, not only in the history of the operation itself, but in the treatment of the disease to which it is applicable."

On the third day of the session Dr. John S. Billings, U. S. A., made an address on Medical Literature. He said:

"There are at the present time, scattered over the earth, about one hundred and eighty thousand medical men, who, by a liberal construction of the phrase, may be said to be educated—that is, who have some kind of a diploma, and for whose edification this current medical literature is produced. Of this number about eleven thousand six hundred are producers of or contributors to this literature, being divided as follows: United States, 2,800; France and her colonies, 2,600; the German Empire and Austro-Hungary, 2,300; Great Britain and her colonies, 2,000; Italy, 600; Spain, 300; all others, 1,000. These figures should be considered in connection with the number of physicians in each country; but this I can only give approximately, as follows: United States, 65,000; Great Britain and her colonies, 35,000; Germany and Austro-Hungary, 32,000; France and her colonies, 26,000; Italy, 10,000; Spain, 5,000; all others, 17,000.

"The 'Index Medicus' for the same year shows by analysis that the total number of medical books and pamphlets, excluding periodicals and transactions, was 1,643, divided as follows: France, 541; Germany, 364; United States, 310; Great Britain, 182; all others, 246. This does not include the inaugural theses, of which 693 were published in France alone.

"The total number of volumes of medical journals and transactions of all kinds was, for the year 1879, 850, and for 1880, 864. The figures for 1880 are too small, but the real increase is slight. During the year 1879, the total number of original articles in medical journals and transactions which were thought worth noting for the 'Index Medicus' was a little over 20,000.

Only a few books older than the present century are, in the opinion of the writer, of any value. The exceptions to the rule are the works of Hippocrates, Galen, Harvey, Hunter, Morgagni, and Sydenham."

Before closing he took occasion to offer the following excellent advice:

"The editors of Transactions of Societies, whether these are sent to journals, or published in separate form, often commit numerous sins of omission in the matter of titles. The rule should be that every article which is worth printing is worth a distinct title, which should be as concise as a telegram, and be printed in a special type. If the author does not furnish such a title it is the editor's business to make it; and he should not be satisfied with such headings as 'Clinical Cases,' 'Difficult Labor,' 'A Remarkable Tumor,' 'Case of Wound, with Remarks.' The four rules for the preparation of an article for a journal will then be: 1st, have something to say; 2d, say it; 3d, stop as soon as you have said it; 4th, give the paper a proper title."

On the fifth and sixth days the general sessions were devoted to addresses by Professor Pasteur and T. H. Huxley, LL.D.

The Congress passed resolutions endorsing vivisection, distributed medals of honor, adopted resolutions of thanks and dissolved.

The selection of the place for the next meeting was left to the executive committee. The social features of the occasion—"the meetings without order"—were greatly enjoyed. The entertainments were abundant and elegant. The hospitality, bountiful and cordial. The Congress was a grand, positive, and permanent success.

